# Game-Based Rehabilitation for Myoelectric Prosthesis Control

**DOI:** 10.2196/games.6026

**Published:** 2017-02-09

**Authors:** Cosima Prahm, Ivan Vujaklija, Fares Kayali, Peter Purgathofer, Oskar C Aszmann

**Affiliations:** ^1^ Christian Doppler Laboratory for Restoration of Extremity Function Department of Plastic and Reconstructive Surgery Medical University of Vienna Vienna Austria; ^2^ Clinic for Trauma Surgery, Orthopaedic Surgery and Plastic Surgery Department of Neurorehabilitation Systems University Medical Center Göttingen Göttingen Germany; ^3^ Department of Bioengineering Imperial College London London United Kingdom; ^4^ Human Computer Interaction Group Institute for Design and Assessment of Technology Technical University of Vienna Vienna Austria

**Keywords:** upper limb prosthesis control, upper extremity amputees, gaming, serious games, neuromuscular rehabilitation, intrinsic motivation, EMG control

## Abstract

**Background:**

A high number of upper extremity myoelectric prosthesis users abandon their devices due to difficulties in prosthesis control and lack of motivation to train in absence of a physiotherapist. Virtual training systems, in the form of video games, provide patients with an entertaining and intuitive method for improved muscle coordination and improved overall control. Complementary to established rehabilitation protocols, it is highly beneficial for this virtual training process to start even before receiving the final prosthesis, and to be continued at home for as long as needed.

**Objective:**

The aim of this study is to evaluate (1) the short-term effects of a commercially available electromyographic (EMG) system on controllability after a simple video game-based rehabilitation protocol, and (2) different input methods, control mechanisms, and games.

**Methods:**

Eleven able-bodied participants with no prior experience in EMG control took part in this study. Participants were asked to perform a surface EMG test evaluating their provisional maximum muscle contraction, fine accuracy and isolation of electrode activation, and endurance control over at least 300 seconds. These assessments were carried out (1) in a Pregaming session before interacting with three EMG-controlled computer games, (2) in a Postgaming session after playing the games, and (3) in a Follow-Up session two days after the gaming protocol to evaluate short-term retention rate. After each game, participants were given a user evaluation survey for the assessment of the games and their input mechanisms. Participants also received a questionnaire regarding their intrinsic motivation (Intrinsic Motivation Inventory) at the end of the last game.

**Results:**

Results showed a significant improvement in fine accuracy electrode activation (*P*<.01), electrode separation (*P*=.02), and endurance control (*P*<.01) from Pregaming EMG assessments to the Follow-Up measurement. The deviation around the EMG goal value diminished and the opposing electrode was activated less frequently. Participants had the most fun playing the games when collecting items and facing challenging game play.

**Conclusions:**

Most upper limb amputees use a 2-channel myoelectric prosthesis control. This study demonstrates that this control can be effectively trained by employing a video game-based rehabilitation protocol.

## Introduction

The initial control of a myoelectric prosthesis can be a frustrating experience, especially after the already traumatic event of losing a limb. Due to the nonintuitive interface, which handles a complex mechatronic system, the cognitive demand for controlling the prosthesis is high and further delays the actual use of the device in everyday life [1,2]. At least 50% of upper extremity amputees report problems with prosthesis control and functionality [3,4], which can be attributed to the need for receiving more training in handling the prosthesis [5,6]. By providing more training opportunities, the user can fully benefit from the technical functions of the prosthesis.

To prepare the residual muscles and induce specific brain plasticity, having access to a prosthesis itself is not necessarily needed. Regaining muscle strength and coordination is a cognitively exhausting and repetitive process, during which the proper execution of movements is reestablished using surface electromyographic (EMG) feedback [2,7,8]. Through physiotherapy, patients are presented with a variety of tasks promoting the development of coping strategies for dealing with the activities of daily living, and introducing the embodiment of the prosthetic system itself. To effectively control their prosthesis, patients need to learn how to properly contract their muscles; the strength of activation and isolation of a single muscle are important parameters [1,9,10].

Although the standard rehabilitation program offers direct functional benefits, its main shortcomings are the lack of motivation for patients to pursue it without the involvement of a therapist throughout the lengthy process. In addition to the loss of functionality, patients may suffer from posttraumatic depression, further decreasing motivation for rehabilitation [11]. Transferring traditional EMG rehabilitation protocols to a virtual setting, and incorporating video games into the training process, can potentially increase the patient’s engagement and perseverance [12]. This approach also provides medical professionals with quantitative data of the patient’s performance.

Many studies report that the progress achieved during rehabilitation based on a playful concept is faster and superior to conservative physiotherapeutic exercises [9,13-15]. These rehabilitation games are especially popular in older adults [16], and when treating patients affected by stroke [17,18] and Parkinson’s disease [19,20]. Various research groups have addressed adding virtual games to an otherwise dull routine in the area of upper limb amputee rehabilitation. There is, however, a difference between virtual or augmented reality environments and using commercially available video games during therapeutic interventions [9,21]. The latter provides greater accessibility and allows patients to easily set up the games at home, and games can be chosen that are proven to motivate the players and maintain engagement over a longer period of time [15]. An example of a commercially available video game that has been interfaced using EMG signals is *Guitar Hero* [14]. This game is based on rhythm and speed, and requires a fast reaction from the player and an immediate transmission of the processed EMG signals to the gaming system. Similarly, a rehabilitation concept for stroke patients using a modified version of the WiiMote control is used for rehab purposes of upper limb amputees, in which EMG signals are matched to the keys of the WiiMote [22]. However, controls are only limited to two motions. Other groups chose a game similar to the arcade classic *Pong,* in which the user’s muscle activity is mapped into a paddle motion that hits a ball into their opponent’s court [23]. Although those approaches can be motivating, the necessary actions are not very intuitive and are not directly transferable to the handling of a prosthesis [24].

This study presents an interface between a computer and a commercially available surface EMG electrode system (Ottobock Healthcare GmbH, 13E200), which is commonly used for controlling prostheses, to evaluate the short-term effects on controllability after a video game-based rehabilitation protocol.

This study, compared to previous studies, prompted participants to not only conduct repetitive agonist and antagonist muscle activation, but also to train and exert sustained contractions over a short period of time, perform precisely timed contractions, and elicit simultaneous contraction of both muscles and muscle groups. These functions are similar to how patients would control a real prosthesis as they interact with their environment.

## Methods

Eleven able-bodied participants, who had no prior experience in EMG control, took part in appraising the benefits of the video game-based training. The categories of the EMG controllability assessments that were evaluated consisted of a provisional maximum voluntary muscle contraction for calibration, precision control, electrode separation, and endurance control by retracing a sine curve with the EMG signal. These assessments are further explained in detail in the *Electromyographic Assessments* subsection. Three video games and their respective control variations were evaluated for their motivational factors and feasibility. Two questionnaires were given to evaluate (1) the video games and the input method, and (2) intrinsic motivation. This study was approved by the ethics committee at the Medical University of Vienna (number 1301/2015) and all study participants read and signed the consent form before taking part.

### Participants

Eleven naïve, able-bodied participants without any known neurological or muscular impairments participated in the study. All participants had normal or corrected-to-normal vision and were instructed and accompanied by the examiner throughout the entirety of the study.

### Experimental Protocol

**Figure 1:**
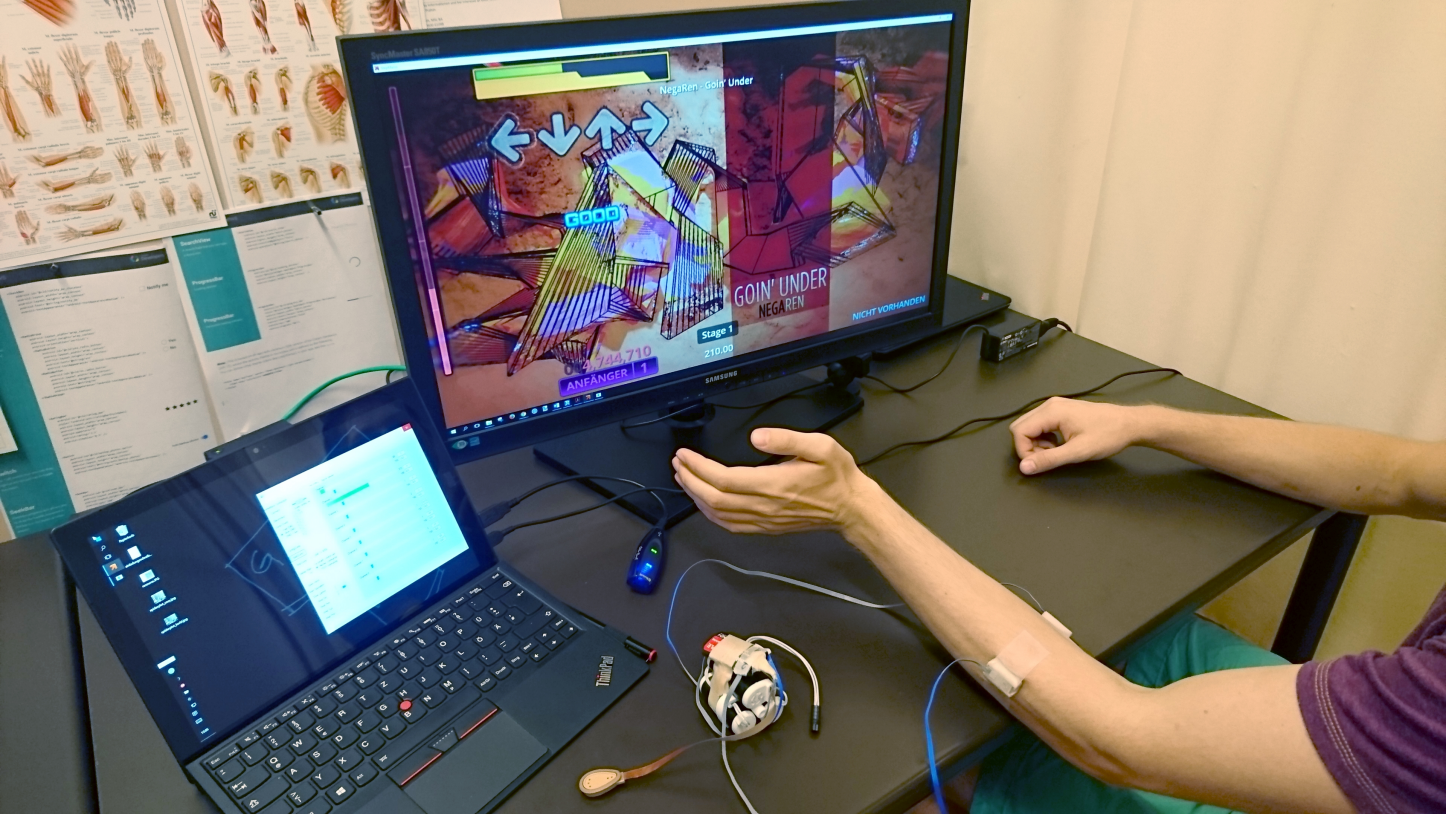
The experimental set-up.

Each participant was seated comfortably in front of two computer screens. One screen displayed the acquired EMG data per electrode channel, the other screen showed the game that the participant was playing. Two active surface EMG electrodes (Ottobock Healthcare GmbH 13E200) were positioned on top of the prominent flexor and extensor muscles of the wrist on the participant’s nondominant side (see Figure 1). This was done to match the handedness of the amputees, which is always transferred on the intact limb regardless of the preimpairment state. Amplification and electrode placement remained the same throughout the sessions. Each electrode delivered root mean square (RMS) at the 100-hertz rate of the recorded EMG signal following the embedded filtering and rectification.

Participants were invited two times and had three test sessions in total: one Pregaming and Postgaming measurement, both conducted on the same day; and one Follow-Up measurement to evaluate short-term retention rate two days later.

Participants were initially instructed to perform three basic EMG assessments: the provisional maximum voluntary contraction (MVC) level, accuracy of electrode control, and muscle endurance. After a short break, participants were presented with three computer games in randomized order. After each EMG-controlled game, they were asked to complete a short user evaluation survey regarding the gaming experience. After the third and final game, a modified questionnaire aiming at intrinsic motivation (Intrinsic Motivation Inventory; IMI) was completed.

### Electromyographic Assessments

**Figure 2:**
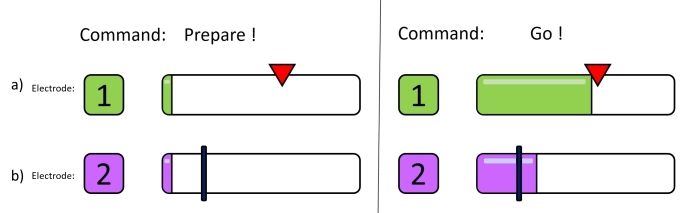
The interface for the precision control assessment and separation of electrode activation. Electrode 1 is used to assess precision control while electrode 2 indicates either the separation or cocontraction of both electrodes. a) The red triangle indicates the goal activation level that participants must reach with their electromyographic signals. b) The black bar marks the threshold at which the electrode is considered active. If the electromyographic signal passed this bar, electrode separation failed and cocontraction is detected.

To investigate the changes in overall controllability, three basic assessments evaluating approximate strength, muscle precision control and coordination, and muscle endurance were performed. The outcome measures that were considered included fluctuations of RMS EMG signals over expected EMG signals. Both electrodes typically show some negligible offset activation (due to common noise) during the idle state of the forearm muscles (see Figure 2).

#### Maximum Voluntary Contraction Test

The MVC test was used as a calibration of the voltage detected by the electrodes, and assessed the MVC force (averaged over 3 trials) for each of the two electrode channels. Participants were asked to maximally contract one muscle and to hold this contraction for 1.3 seconds, of which only the last second was taken for calculating the activation baseline.

#### Assessment of Precision Control

The Assessment of Precision Control test evaluated the participant’s fine EMG control accuracy. The range of this test was adapted based on the outcome of the MVC test. For each electrode, the participant was asked to reach 30 randomly preselected activation levels in the range of 10-90% MVC, and sustain them for 300 milliseconds each. The required level of activation was indicated by a triangular mark on the EMG bar (see Figure 2 a). A total of 30 marks (3 trials consisting of 10 levels) were performed for each electrode. The percentile deviation from the mark was taken as outcome measure. Randomization of the goal activation marks took place once before the beginning of the study and was kept constant between all participants.

#### Assessment of Separation

The Assessment of Separation test is a subsection of the Assessment of Precision Control, and determined whether participants could separately control one muscle or if the opposing electrode was activated (cocontraction) during the tasks of the Precision Control assessment. Depending on the MVC, a threshold was set that corresponded to the point of EMG activation at which an electrode was considered active (see Figure 2 b). This threshold was set at 15% MVC. The concept of reaching a certain threshold for activating the electrode corresponds to the actual execution of prosthetic movements [1,25]. The outcome measure was the binary activation of the opposing electrode and the overall percentage of activation of the opposing electrode per participant.

#### Assessment of Endurance Control

The Assessment of Endurance Control test assessed muscle coordination and muscle fatigue while the participants used their EMG signals to closely follow a sine curve (1/4 hertz) on the screen until they felt fatigued. The estimated force needed to reach the peaks of the sine curve corresponds to 60% MVC. A positive value corresponded to activation of the first electrode, while a negative value corresponded to the second. Electrode activation needed to be separate to reach the peaks of the sine curve. The minimum time to be reached in this test was 15 minutes. The outcome measure was the EMG signal deviation from the desired sine curve, given as correlation r² [26].

### Games

**Figure 3:**
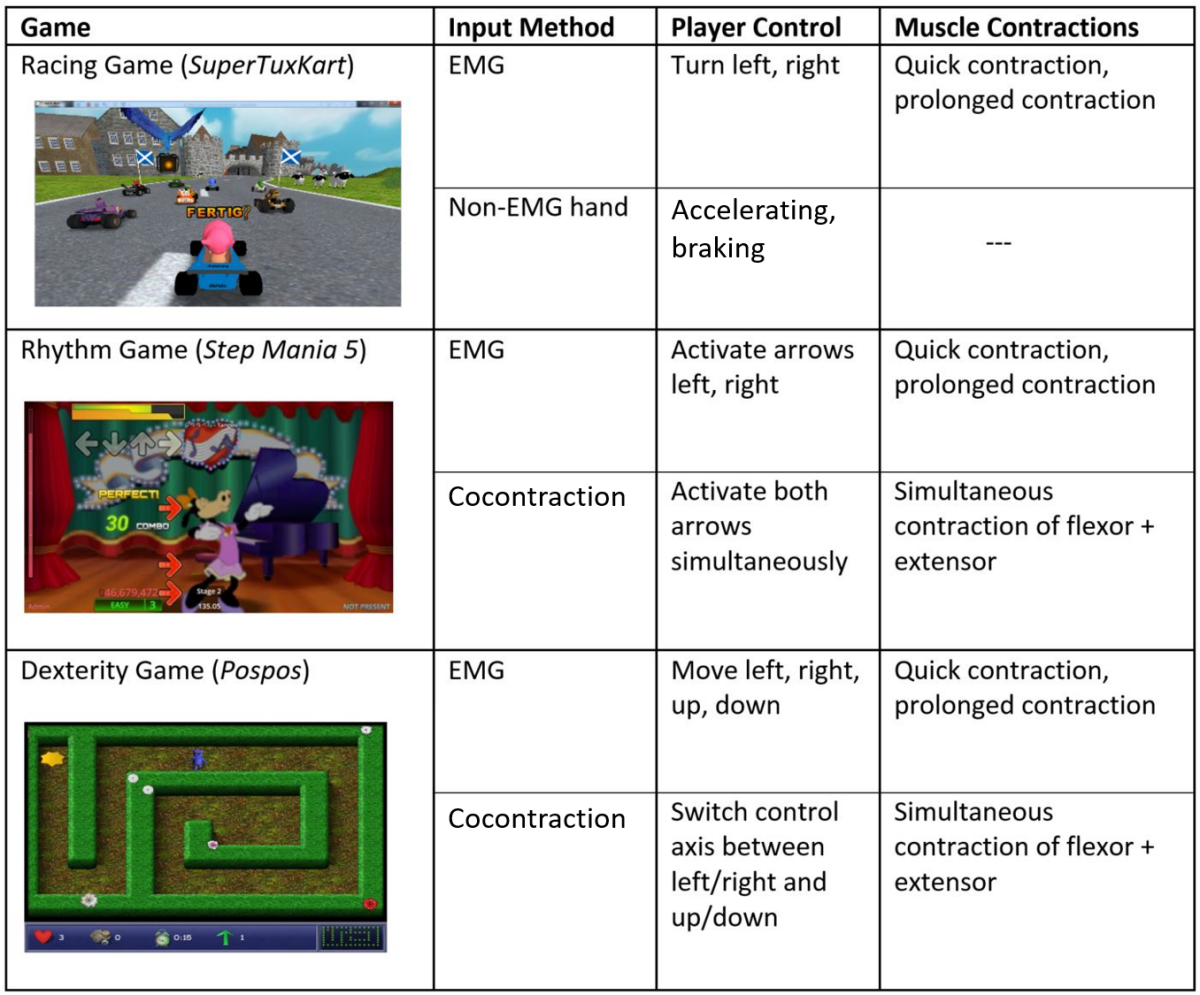
An overview of the three games played by the participants, with their respective input methods and muscle contraction types needed to drive the game. EMG: electromyographic.

Three different open-source games were used in this study: a racing game, a dexterity game, and a rhythm-based game. Each game featured its own respective control method (see Figure 3). The general input mechanism was to substitute keyboard events with EMG activation. Participants controlled two electrodes, making two concurrent keys possible at a time. Those motions represented one degree of freedom (DoF). However, to allow for more than just one DoF steering, participants could perform a cocontraction (a quick simultaneous activation of both opposing muscles) and switch to a second DoF. For games that did not offer the option of cocontraction to switch through keys, the dominant limb supported the control through direct keyboard input.

#### Racing Game

In the 3-dimensional racing game *Super Tux Kart* [27] the player raced against the clock and computer-controlled adversaries. The participant controlled left and right turns solely with EMG signals, whereas accelerating and braking were controlled using keyboard inputs with their dominant hand. Required EMG activations were quick contractions and sustained contractions.

#### Dexterity Game

In the dexterity game *Pospos* [28] the player had to maneuver through a 2-dimensional labyrinth and collect items. This game was controlled entirely through the participant’s EMG signals. Switching between the DoFs was done by cocontraction, which corresponded to controlling the horizontal and vertical axes of the player. Required EMG activations were quick contractions, sustained contractions, and cocontractions to switch between DoFs.

#### Rhythm Game

In the rhythm-based game *Step Mania 5* [29] participants were prompted to activate 2 different arrow-shaped buttons using their EMG signal. The arrows had to be quickly pressed or held in time matching the rhythm of the note patterns that scrolled across the screen. Required EMG activations were quick contractions, sustained contractions over a certain time period, and cocontractions to simultaneously activate two buttons.

### Questionnaires

Participants were given two questionnaires to complete: (1) a modified IMI questionnaire; and (2) a user evaluation survey about the EMG assessment, the games that were played, and control methods.

#### Modified Intrinsic Motivation Inventory Questionnaire

A modified 28-item version of the IMI [30-32] consisting of five subscales was used to evaluate the experience with the video games that were played. The five subscales formed scores for enjoyment, perceived competence, perceived choice, pressure felt, and immersion. An additional six questions were added to the last subsection to evaluate immersion into the games. The questionnaire was adapted to the study by changing the words, “working” and, “doing” to, “playing”. The questionnaire included statements such as, “I found the games very interesting” and, “I felt tense while playing.” The statements were rated on a 7-point Likert rating scale ranging from 1 (*no, not at all*) to 7 (*yes, definitely*).

#### User Evaluation Survey

The user evaluation survey consisted of (1) rating the games that were played, (2) rating the input and player control methods (see Figure 3), (3) rating the EMG assessment, and (4) identifying engaging elements within each game. This short survey about gaming experience was presented after every game and included questions about the gameplay, fun factor, motivation, and input and control methods.

### Statistical Analyses

All analyses were conducted using IBM SPSS 20 and Matlab 2013b. Nonparametric tests were performed on data not meeting the requirement for normal distributions. Normal distributions were assessed via graphical interpretation showing normal Q-Q plots, and with Shapiro-Wilk tests for normality as a numerical assessment. Significance was set at Cronbach alpha=.05.

#### Controllability

##### Maximum Voluntary Contraction

This test was used to relatively set the maximum contraction limit for the subsequent EMG tests, and was given as average of the RMS electrode activation. MVC was measured three times: before playing the games, directly after playing the games, and before the Follow-Up measurement.

##### Assessment of Precision Control

The outcome measure for this test was the percent deviation from the 30 goal points per electrode channel. The deviation from the goal was calculated in absolute values and set in relation to the achieved MVC to derive a percent value of deviation for each goal point. All 30 data points were treated as if they were performed consecutively. The goal points were divided into three equidistant intensity sections ranging from 10% MVC to 90% MVC. The Shapiro-Wilk test confirmed a normal distribution with *P*<.001. The mean and standard deviation were compared for significance for the activation levels (low, middle, high) and for measurement sessions (Pregaming, Postgaming, Follow-Up) with a Bonferroni corrected paired samples t-test.

##### Assessment of Separation

Threshold crossings were given in percentages for each of the 3 goal activation levels over the 3 measurement sessions. The three equidistant intensity levels ranged from 10-90%. Improvement was tested for significance with a related samples Wilcoxon signed rank test.

##### Assessment of Endurance Control

The conformity of retracing the sine curve as a correlation r² was computed for defined time windows consisting of 30 seconds. The highest r² value was taken from a period of at least 200 seconds. A related samples Wilcoxon signed rank test (Cronbach alpha=.05) examined the Pregaming measurement correlation with the Follow-Up measurement correlation.

#### Questionnaires

##### Intrinsic Motivation Inventory Questionnaire

Participants had to rank the 28 statements from 1 to 7, where 1 represented *I do not agree* and 7 represented *I agree*. The statements belonged to one of five categories and the ranking was averaged. An independent samples Mann Whitney U test was performed to describe the data. All categories except *pressure* had a high desirable rank.

##### User Evaluation Survey

This survey consisted of ranked statements on a 5-point scale, and multiple choice questions regarding game experience and preferences that were evaluated via a frequency analysis.

## Results

### Controllability

#### Maximum Voluntary Contraction

The MVC was used only as a calibration for the electrode channel voltage. However, it could be observed that the RMS values for the MVC test directly after the games showed an increase instead of the expected decrease. Moreover, the same can be observed for the Follow-Up session.

#### Assessment of Precision Control

**Figure 4:**
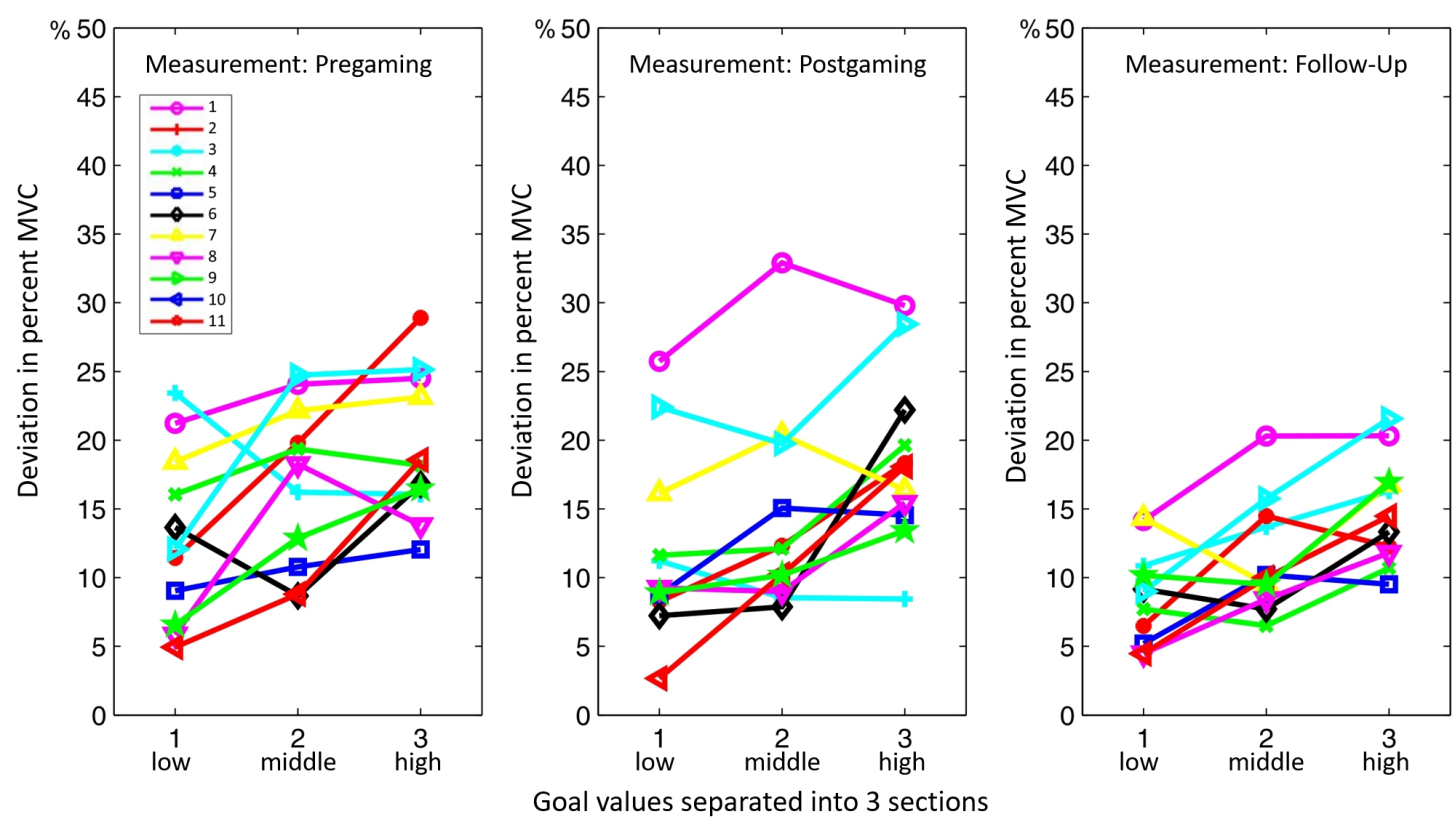
Development of the deviation of the electrode activation around the goal value levels (separated into low, middle, and high goal values) through all three measurement sessions (Pregaming, Postgaming and Follow-Up). MVC: maximum voluntary contraction.

Results showed a significant improvement in fine accuracy and electrode coordination from the Pregaming measurement to the Follow-Up session (*P*=.001). Percentile deviation from the goal value was high and heterogeneous in the first two measurements (Pregaming and Postgaming), but significantly lower and more homogeneous in the Follow-Up measurement (*P*=.001; see Figure 4). In all 3 measurement sessions, it was significantly harder to reach a high goal activation level compared to a low one (*P*=.002).

#### Assessment of Separation

**Figure 5:**
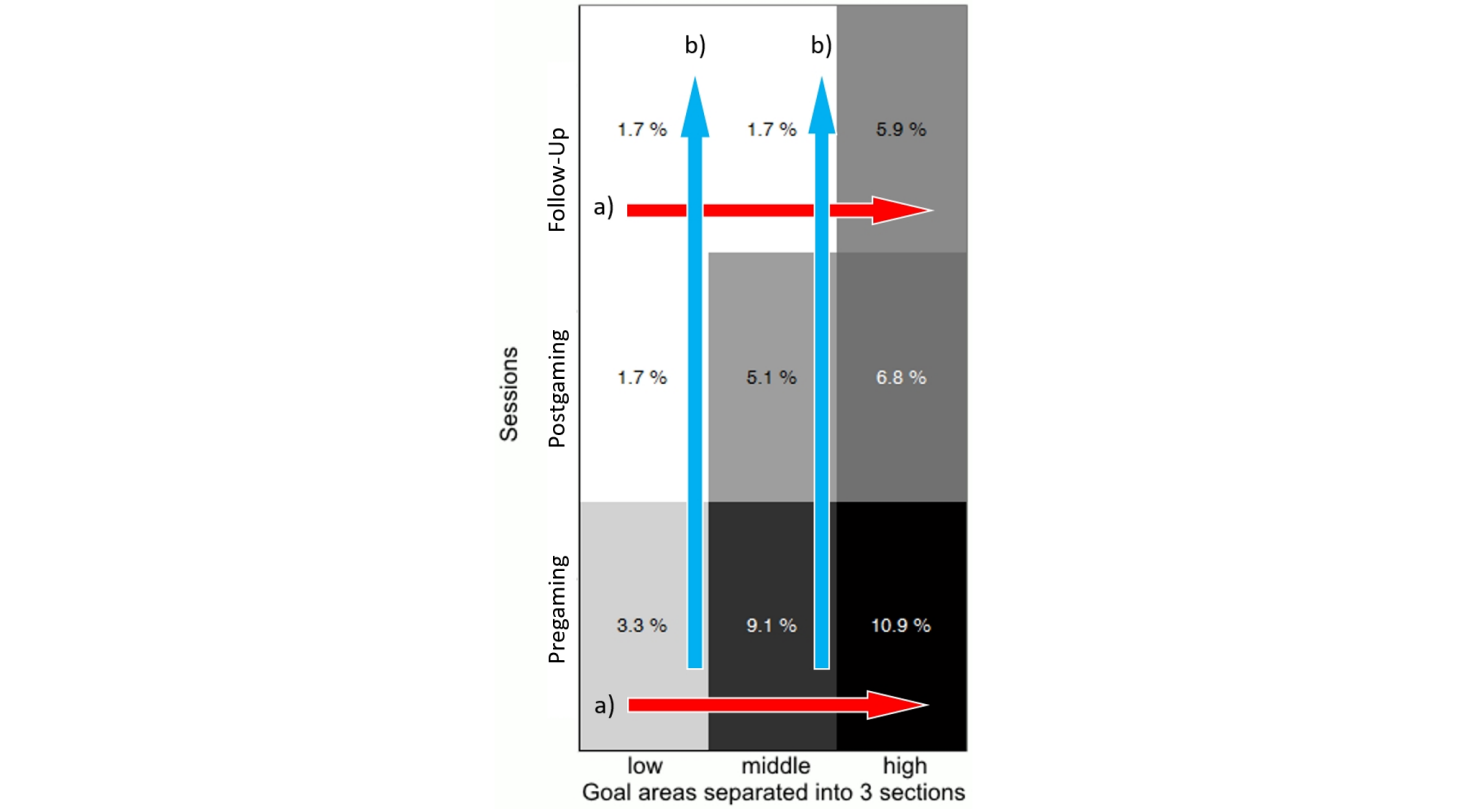
Opposing electrode activations over three measurement sessions (Pregaming, Postgaming and Follow-Up) and three goal activation areas, which the participants had to reach with their electromyographic signal (low, middle, and high). a) Comparison within the session. b) Comparison between the sessions.

Significantly better performance could be observed during low level electrode activation tasks compared to high goal activation tasks within Pregaming (*P*=.02) and the Follow-Up session (*P*=.04; see Figure 5 a). There was a significant decrease in opposing electrode activation from the first to the last measurement session for low (*P*=.04) and middle (*P*=.02) goal activation levels; however, this was not true for high intensity goal activation (see Figure 5 b). There was a significant improvement in electrode separation overall, considering the first and last measurement sessions (*P*=.02).

#### Assessment of Endurance Control

**Figure 6:**
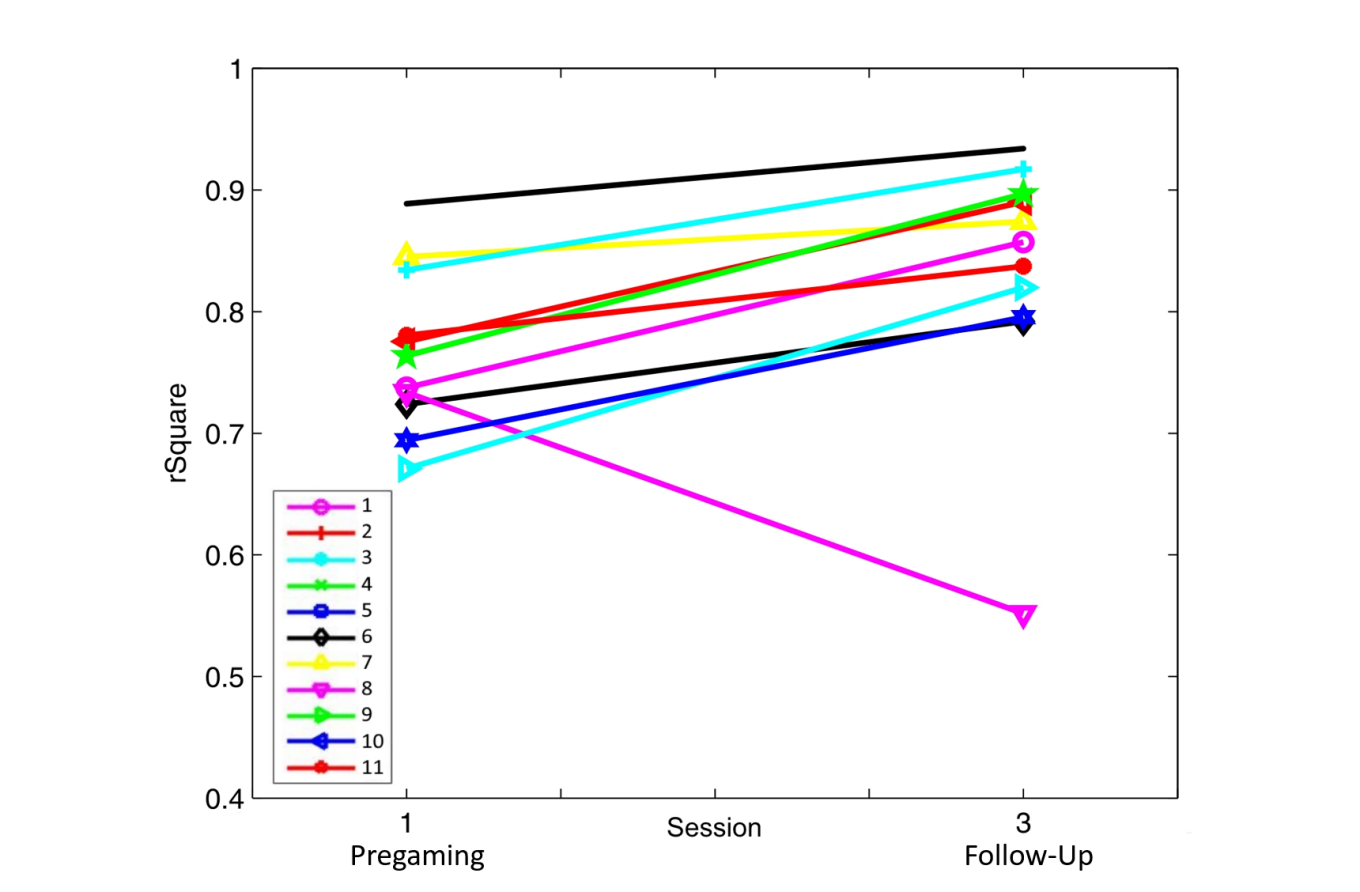
Scores of the endurance assessment and comparison of Pregaming r² value to the Follow-Up value. High r² corresponds to close electromyographic retracing of the given sine curve.

Participants showed an improvement in muscle endurance control (see Figure 6). This result was true for all but one participant, whose EMG activation expressed an offset and was shifted by half a period of the retraced sine curve. Nevertheless, the Related Samples Wilcoxon Signed Rank Test determined a significant difference (*P*=.004) in r² performance between the Pregaming and Follow-Up measurement sessions.

### Questionnaires

#### Modified Intrinsic Motivation Inventory

**Figure 7:**
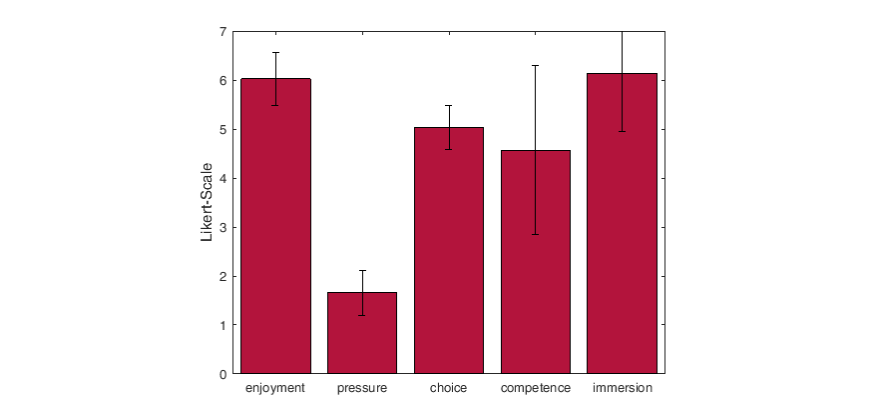
Means and standard deviations for 5 subscales of the modified intrinsic motivation questionnaire on a scale from 1 (low) to 7 (high).

Results obtained from the IMI questionnaire can be viewed as mean and standard deviation of the five categories in Figure 7. Participants enjoyed playing the games and felt immersed when doing so. Participants perceived playing the games as their own choice and felt competent and at ease while doing so. *Pressure* was the only category in which a low rank was desirable.

#### User Evaluation Survey

**Figure 8:**
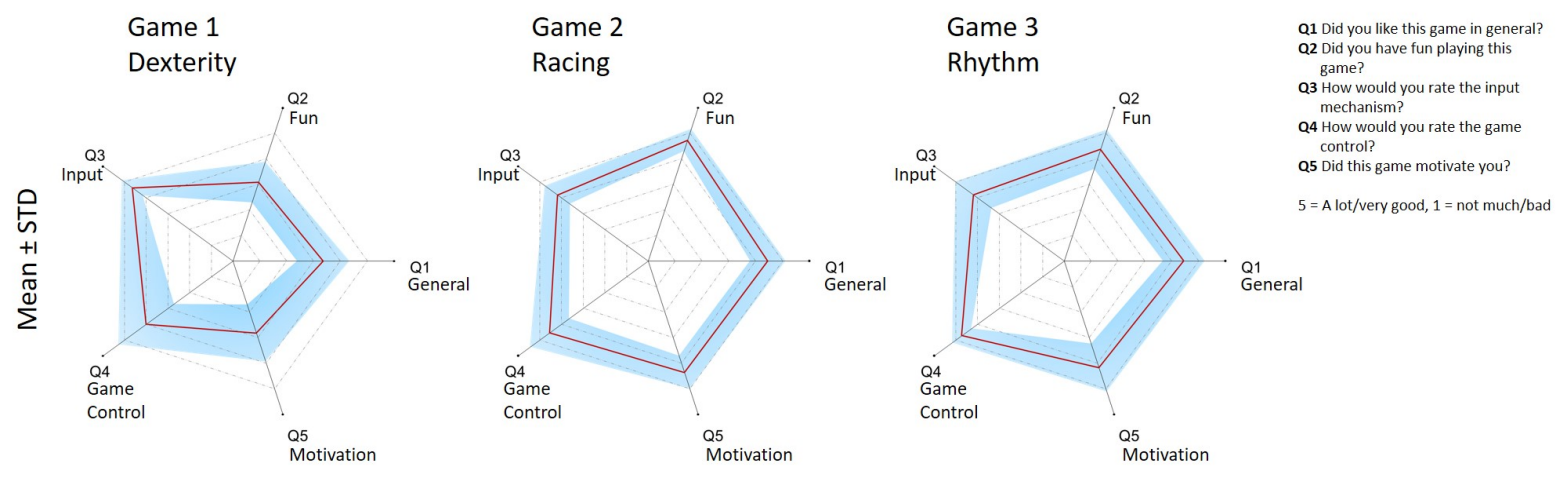
Mean and standard deviation ratings of the three games played, according to the survey that participants had to fill in after each game.

Participants were asked to answer five questions for each game (Q1-Q5; see Figure 8). According to the user evaluation survey, the favorite game (derived from the score of Q1 and Q2) was the racing game *Super Tux Cart* followed by the rhythm game *Step Mania 5*. According to Q3 and Q4, participants preferred to control the games with EMG signals only and to perform different contraction lengths as well as cocontractions. In terms of motivation (Q5), the *Pospos* dexterity game ranked far behind the racing and rhythm games, which were equally well received.

The most important components to ensure continued play and enjoyment of a game were (listed according to importance): (1) the EMG control method, (2) the level of difficulty, (3) dynamic movements, and (4) collecting items. Music, atmosphere, and graphics rated last. Although participants claimed to prefer 3-dimensional graphics, this finding did not reflect their rating of what they enjoyed most in the games. The most motivating aspects of games were (1) the gameplay, (2) to see one’s own high score, and (3) to clear upgrades.

**Figure 9:**
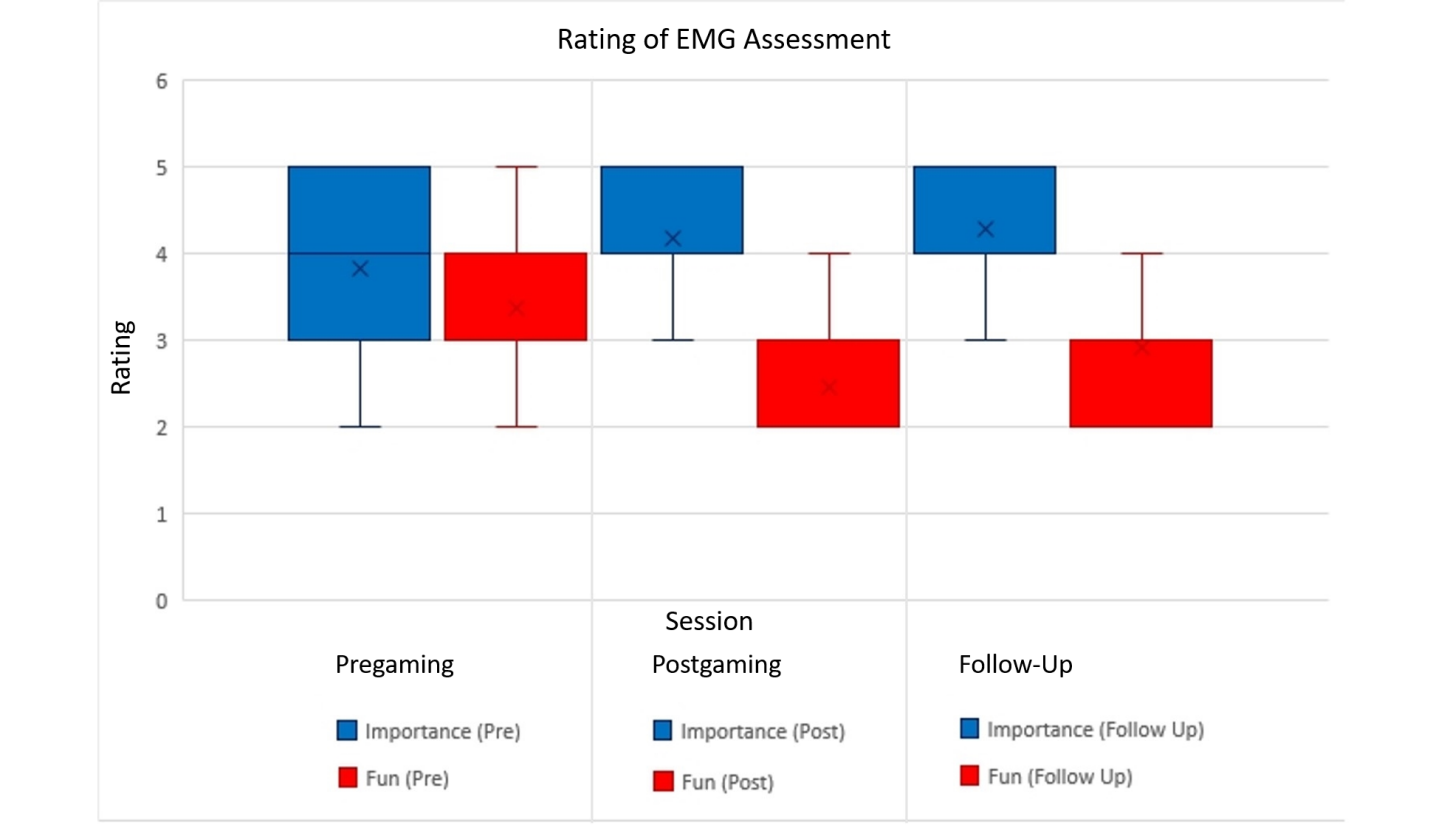
Mean and standard deviation for ratings of the electromyographic assessment after each session, ranging from 5 (very important/fun) to 1 (very unimportant/boring). EMG: electromyographic.

Additionally, participants had to rate the EMG assessments after each session. Participants were asked about how important they thought the EMG assessments were, and to rate the fun they had while doing them. As can be seen in Figure 9, rating of the importance of the EMG assessment increased until the Follow-Up measurement (however, not significantly), while the participants enjoyed them significantly less (*P*=.002). Interestingly, a slight rise in rating the fun factor was observed after the Follow-Up session.

## Discussion

Results from this study demonstrate improvements in fine accuracy electrode activation and electrode separation from Pregaming EMG assessments to the Follow-Up measurements. Surprisingly, the MVC values used as a baseline calibration also showed an increase, instead of the expected decrease, after playing the games. This result could be due to either warmth or sweat that would influence the electrode resistance. Additionally, this result is a strong indicator that the gaming session was not fatiguing for the participants. Performance during the precision control assessment, however, declined after playing the games. If, based on previous investigations, we exclude fatigue, it is reasonable to assume that participants started losing their concentration by the end of the sessions. In the Follow-Up measurement, a clear improvement in performance was observed, which can be attributed to the restoration of full focus combined with the obtained experience from the previous session.

Compared to previous studies [22-24,33], participants not only conducted repetitive flexor and extensor muscle activation, but also sustained contractions over varying periods of time, performed precisely timed contractions, and executed simultaneous contractions of both muscle groups. These actions are similar to how patients would control a real prosthesis.

The motivational aspects of training gamification are clear, and are likely the main advantage compared to conventional techniques. It is reasonable to assume that certain improvement of the EMG control could be observed by sole application of the listed EMG tests. However, prolonged exposure to such stimuli would certainly lead to a loss of interest, which is sure to be maintained by the appealing context of a video game [15].

### Limitations

The transferability of the obtained results to the amputee population might be questioned, since this study was conducted strictly with healthy participants. However, based on the outcomes reported in other myocontrol-based studies [34,35], it is reasonable to expect that the patient group would perform similarly.

This study was a short-term intervention, and can be viewed as a proof of concept. Further research will incorporate a long-term evaluation of video game-based interventions, as well as additional exploration of advanced control mechanisms, such as those based on machine learning approaches [36-38].

### Conclusion

Most upper limb amputees use a 2-channel myoelectric prosthesis control. This study demonstrates that this control can be effectively trained by employing a video game-based rehabilitation protocol. Participants significantly improved their electrode separation and fine muscle control. It could be shown that the enjoyment of the games was greater than that of the EMG assessments, which decreased over time. Additionally, engaging elements within each game could be identified. A subsequent study with an amputee population will show if the information gained from healthy participants can be transferred to patients. The final outcome would be a robust system that patients can operate outside of a clinical environment.

## Abbreviations

DoF: degree of freedom
EMG: electromyographic
IMI: Intrinsic Motivation Inventory
MVC: maximum voluntary contraction
RMS: root mean square
